# Circadian clock gates diurnal glucose utilization

**DOI:** 10.1371/journal.pbio.3003743

**Published:** 2026-04-17

**Authors:** Yao D. Cai, Joanna C. Chiu

**Affiliations:** Department of Entomology and Nematology, College of Agricultural and Environmental Sciences, University of California Davis, Davis, California, United States of America

## Abstract

The circadian clock and cellular metabolism are tightly coupled. This primer discusses a recent PLOS study that elucidates how glucose metabolism changes over the course of the day in Drosophila and how this is disrupted in clock and sleep mutants.

## Teaser

The circadian clock and cellular metabolism are tightly coupled to maintain homeostasis. A new study in *PLOS Biology* leverages metabolic tracing to reveal time-of-day-dependent activities of glucose metabolic pathways in *Drosophila* that are disrupted in clock and sleep mutants.

Chrononutrition is at the forefront of modern nutritional science and precision medicine (reviewed in [1]). It centers on the alignment of meal timing with natural circadian (~24-hour) rhythms of metabolism and physiology to optimize health and fitness. This approach is highly intuitive since circadian clocks are known to regulate many aspects of cellular metabolism (reviewed in [2]). At the molecular level, previous studies have established that time-of-day activities of primary metabolic pathways are orchestrated by clock-controlled oscillations in mRNAs, proteins, and even protein modifications. At the metabolite level, there is also ample evidence of temporal regulation. For example, the post-meal blood sugar level is higher in the afternoon as compared to morning measurements despite the consumption of the same amount of food [3]. More recently, comprehensive metabolite analyses via unbiased and targeted metabolomics have revealed that steady-state abundance of hundreds of metabolites similarly exhibit diurnal rhythms (e.g., [4]). However, measurement of steady-state metabolite concentration is only a snapshot and does not necessarily reflect metabolic flux [5]. This concept should be intuitive to city commuters. A high concentration of cars on the road can represent both high flux when traffic is smooth or zero flux during a traffic jam. Whether metabolic flux exhibits daily rhythms remains largely unknown.

A new study published in *PLOS Biology* by Malik and colleagues revealed time-of-day flux of glucose-derived pathways [6]. By leveraging isotope tracing techniques, they provided evidence for diurnal glucose utilization with a peak in the morning in *Drosophila*. They identified diurnal patterns of glucose metabolic pathway flux, providing new insights that could not be revealed by previous studies that only captured steady-state metabolite abundance.

**Fig 1:**
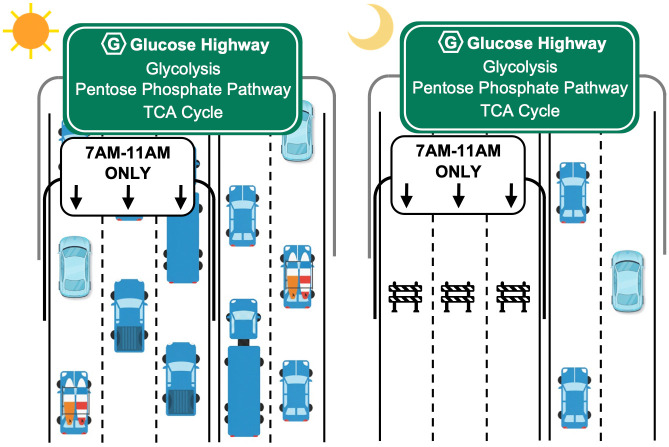
Clock-gated morning rush hour of glucose utilization depicted as highway traffic. In the morning (left), clock-controlled food intake at dawn results in high glucose concentration in *Drosophila* body tissues, analogous to higher number of vehicles on the highway. This increased “traffic flow”, defined by the total number of vehicles passing a given point in a given time, is aided by the opening of the “7AM-11AM only” lanes that are gated by the circadian clock. This is analogous to higher metabolic flux in the morning hours in fly tissues. In other times of the daily cycle (right), both glucose concentration (number of cars) and pathway flux (traffic flow) are low. Vehicle icons were generated by authors using Google’s Nano Banana 2 from the prompts such as “create a top-view of a car icon in white background”, February 16, 2026.

The authors first examined daily variations of plasma metabolites in pathways downstream of glucose metabolism in healthy humans. These data were then used to establish an analytical framework to profile metabolic flux rhythms in *Drosophila* using isotope tracing of ^13^C_6_-glucose. They observed a robust morning flux into downstream pathways, which they termed “rush hour” (Fig 1). This morning flux funnels glucose into glycolysis, the pentose phosphate pathway (PPP), tricarboxylic acid cycle (TCA) cycle, and amino acid biosynthesis. This rush hour follows peak timing of food intake of flies at dawn, which likely results in a morning peak of glucose 6-phosphate [7], the metabolic hub of glycolysis and PPP. Therefore, this temporal pattern is in agreement with previous findings that metabolite concentration positively correlates with metabolic flux [8]. The authors also found the glycogen pool remained stable, suggesting glycogen storage cannot be the sole explanation for the observed diurnal metabolic flux. The next obvious question the authors addressed is whether circadian clocks anticipate the rush hour and promote the morning flux. This could perhaps be enabled by an increase in clock-controlled expression of genes involved in glucose metabolism prior to the rush hour.

Utilizing well-characterized circadian clock mutants in *Drosophila*, the authors reported the rush hour of glucose utilization is attenuated when the circadian clock was abolished. In contrast, flies experiencing short-term fasting still exhibit the rush hour. These data suggest circadian clocks indeed contribute to shaping the diurnal flux of glucose utilization, independent of food intake. As diurnal animals, flies are physically more active in the daytime, and activity is also under clock control. Therefore, circadian clocks may optimize the timing of food intake and utilization of glucose with diurnal energy demand.

In modern society, diurnal energy demand can be altered due to shift work, exercise, and disease state. The authors then asked whether animals maintain diurnal glucose metabolism regardless of diurnal energy demand. They leveraged a hyperactive *Drosophila* mutant with defective dopamine signaling, *fumin* [9]. Note that *fumin* retains a functional endogenous clock, thus allowing the assessment of diurnal energy demand on glucose metabolism without altering circadian clocks. In contrary to wild-type flies, *fumin* mutants exhibit two rush hours of glucose utilization. The gained rush hour at dusk is distinctive from the morning rush hour. Furthermore, TCA cycle becomes the preferred pathway, which correlates with high energy demand of the mutant. Peak glycogen hydrolysis in the morning in *fumin* mutants further supports clock-controlled glucose utilization, regardless of high energy demand throughout the day. Whether the clock-independent reorganization of daily glucose flux is general to hyperactivity or specific to altered dopamine signaling pathways remains to be explored. Curiously, given the short-sleep nature of *fumin* mutants, the role of sleep in regulating daily glucose flux also warrants future investigation.

As the authors noted, the rush hour discovered here represents a metabolic challenge condition, rather than under physiological condition. This is due to technical limitation that a supraphysiological dose of labeled glucose is required for ^13^C isotope detection. Evaluating dose scaling and/or alternative delivery paradigms may further reveal the metabolic flux of glucose utilization under physiological conditions.

To date, profiling of diurnal pathway flux via metabolic tracing is remarkably sparse, highlighting the significant advance of this study. In contrast to steady-state metabolite profiling via metabolomics and targeted biochemical approaches, metabolic tracing provides a direct read-out of pathway activities. Similarly, we have gained insights into the dynamics of other biological processes from capturing flux, as seen in RNA flow analysis across life cycle of transcripts [10].

In summary, Malik and colleagues established a framework for understanding the temporal regulation of metabolic flux, the primary measures of metabolism. Future works could examine the temporal flux of other metabolic pathways, such as gluconeogenesis, and one-carbon metabolism. Combining respirometry and calorimetry with metabolic tracing is expected to reveal a more complete picture of metabolic flux [11]. In addition, cell-type and organ-specific metabolic tracing are expected to reveal in situ metabolic activities and metabolite transport across tissues with higher spatial resolution. Genetically encoded fluorescent reporters, imaging mass spectrometry, and cell-type-specific metabolomics may also help to establish new analytical frameworks for metabolic tracing, as conceptually demonstrated here in the use of steady-state metabolite measurement prior to metabolic tracing. The rapid iteration of deep learning and other artificial intelligence tools will certainly aid the interpretation and integration of these complex systems (e.g., [12]). The accumulative understanding of diurnal metabolic processes is expected to further direct the timing of pharmacotherapy and lifestyle interventions such as diet and exercise to optimize metabolic homeostasis.

## Abbreviation:

PPP: pentose phosphate pathway
TCA: tricarboxylic acid cycle
